# Association between wheeze and selected air pollution sources in an air pollution priority area in South Africa: a cross-sectional study

**DOI:** 10.1186/1476-069X-13-32

**Published:** 2014-05-06

**Authors:** Joyce Shirinde, Janine Wichmann, Kuku Voyi

**Affiliations:** 1 Department of Environmental Health, Tshwane University of Technology, Private Bag X680, Pretoria 0001, South Africa; 2 School of Health Systems and Public Health, Health Sciences Faculty, University of Pretoria, P.O. Box 667, Pretoria 0001, South Africa

**Keywords:** Wheeze, Asthma, Environmental tobacco smoke, Heating fuel, Cooking fuel, Traffic, Air pollution, South Africa, Industrial

## Abstract

**Background:**

An association between wheeze (a symptom of asthma) and environmental tobacco smoke (ETS), types of fuel used for residential heating or cooking and the frequency of trucks passing near homes, has been reported mainly in developed countries. Little is known about the strength of such associations in developing countries. This study was conducted in residential areas situated in Ekurhuleni Metropolitan Municipality, namely Tembisa and Kempton Park, which form part of the Highveld region, a priority area in terms of air pollution in South Africa.

**Methods:**

From 3764 eligible school children, aged between 13 and 14 years, from 16 selected high schools in the study area, 3468 completed a modified questionnaire based on the International Study of Asthma and Allergies in Childhood (ISAAC). Data were analysed using multiple logistic regression models.

**Results:**

The results are based on data from 3424 children. In the adjusted models, exposure to ETS at school was associated with wheeze ever (OR 1.22 95% CI: 1.03 - 1.45) and current wheeze (OR 1.33 95% CI: 1.08 - 1.64). When gas was most frequently used for residential heating the likelihood of wheeze ever increased by 47% (OR 1.47 95% CI: 1.15 - 1.88). Trucks passing near homes for almost the whole day during weekdays, increased the likelihood of wheeze ever (OR 1.32 95% CI: 1.01 - 1.73), current wheeze (OR 1.61 95% CI: 1.15 - 2.24) and current severe wheeze (OR 2.22 95% CI: 1.28 - 3.77). When data were stratified according to residential area, for children living in Tembisa, ETS exposure at home was associated with current wheeze (OR 1.36 95% CI: 1.06 - 1.77); gas most frequently used for residential heating was associated with wheeze ever (OR 1.68 95% CI: 1.23 - 2.28) and current wheeze (OR 1.61 95% CI: 1.08 - 2.39); paraffin most frequently used for residential heating was associated with current severe wheeze (OR 1.85 95% CI: 1.04 - 3.28).

**Conclusion:**

It was concluded that children living in one of the air pollution priority areas of South Africa, have an increased risk of wheezing due to exposure to both indoor and outdoor air pollution sources.

## Background

Environmental air pollution is a major global health risk factor. Studies conducted in various parts of the world have reported a wide range of adverse effects of ambient air pollution [1,2]. There is growing evidence linking respiratory symptoms in children to air pollution [1]. Children are more vulnerable as their immune system and lungs are not fully developed when air pollution exposure begins [3]. Personal exposure of children to air pollutants occurs mainly in three microenvironments, home, school and during transport, which Ashmore and Dimitroulopoulou discuss in detail [3]. Environmental tobacco smoke (ETS), combustion of fuels for residential cooking/heating and the frequency of trucks passing near homes have all been associated with respiratory diseases in children [4,5].

Tobacco smoke is one of the most common indoor air pollutants. As early as the seventies, literature has periodically reviewed ETS, or passive smoking and health [5]. Although parental smoking is the most common source of exposure to ETS, children are also exposed in areas such as schools, restaurants and public transport vehicles [6].

The burning of biomass fuel (BMF) (wood, charcoal, dung, crop residues and other raw plant material), for cooking, heating or both, remain the most widespread and important source of exposure to air pollution [7]. About 2.4 billion people worldwide live in households where BMF is the primary fuel for cooking, heating or both, with more than 90% being in rural areas [7]. Other sources of indoor air pollution are stoves and heaters using gas or paraffin fuel. Gas space heaters have nitrogen dioxide (NO_2_) emission rates similar to gas stoves and are often used for long periods of time in living and sleeping areas. This may result in NO_2_ concentrations four or more times higher than gas stoves used for cooking [8]. Pollutants emitted during paraffin (kerosene) combustion include carbon dioxide, sulphur dioxide, NO_2_, particulate matter, formaldehyde, various hydrocarbons and volatile organic compounds [9].

Exhaust emissions are an important source of traffic-related air pollution. According to the latest World Health Organization (WHO) technical report, there is sufficient evidence linking vehicle emissions to the health of people living in close proximity to roads [1,10]. Many epidemiological studies in developed countries have investigated the association between asthma symptoms (e.g. wheeze) and traffic-related pollution [11-14]. Little is known about the strength of such associations in developing countries, such as South Africa.

Our study was conducted in Tembisa (a township with formal and informal housing) and Kempton Park (a suburb with formal housing), situated in the northern region of Ekurhuleni Metropolitan Municipality (EMM), in the eastern region of Gauteng Province, South Africa. This is the first study using the International Study of Asthma and Allergies in Childhood (ISAAC) methodology, conducted in Gauteng Province, a heavily industrialised urban area. Two previous South African studies applied the ISAAC methodology; one in Cape Town, Western Cape Province, an industrial coastal city; and the second in Polokwane, Limpopo Province [15,16]. It was justifiable to conduct this study in EMM as the meteorology and air are different from that of Cape Town and Polokwane. Ekurhuleni Metropolitan Municipality covers approximately 1923 km^2^ and forms part of the Highveld region, which was the second region in South Africa to be declared an air pollution priority area by the Minister of Environmental Affairs in 2007, in terms of the National Environmental Management: Air Quality Act, 2004 (Act No. 39 of 2004) [17].

According to a baseline air quality assessment report for the EMM in 2004, vehicular exhaust emissions (both petrol and diesel) were identified as significantly contributing to air pollution [18]. Other sources of air pollution included: residential fuel burning (particularly coal), industrial and commercial fuel burning (coal-fired boilers in close proximity to residential areas), OR Tambo International Airport (contributing a small fraction of low level, concentrated NO_2_) and large industries associated with various stack, vent and fugitive emissions [18].

The aim of our study was to investigate the association of wheeze, a symptom of asthma with indoor and outdoor air pollution sources, specifically ETS, the types of fuel most frequently used for residential cooking or heating, transportation to school and the frequency of trucks passing near homes in urban areas of Tembisa and Kempton Park.

## Methods

### Study area

Tembisa is the second largest township in Gauteng Province, with both formal and informal housing and mainly inhabited by people belonging to Black/African ethnic groups. Under apartheid, South Africans were categorised into one of four socially defined race or ethnic groups: African/Black (descent primarily from one of a number of Black language groups in Southern Africa), Coloured (general grouping, including a mixture of Black, Malay, European and indigenous Khoisan ancestry), White (mainly European ancestry) and Asian (Indian sub-continent ancestry). Race is still linked to both past and present access to resources, socio-economic status and educational status. Kempton Park is a suburban area surrounded by industry and arterial roads connecting Gauteng Province. OR Tambo International Airport, which is Africa’s busiest airport, is also located here. Kempton Park residents are predominantly White and it is only in recent years, after the 1994 democratic elections, that some mostly middle income Black/African families moved into the area. According to the Statistics South Africa (2011), most households in Tembisa had an annual household income of between R19 601- R38 200 in contrast Kempton Park where most households had an income of between R153 801 - R307 600 [19].

### Study design, population and sample selection

A cross-sectional epidemiological study was conducted between February and June 2012. The ISAAC Phase I protocol was followed [20]. A list of all schools (primary and secondary) in EMM was provided by the Gauteng Department of Education and 16 high schools were randomly selected from this list. Each school was contacted and requested to participate in the study. Following the approval of the study by the principal and governing body in each school, all eligible children between the ages of 13 and 14 years and in Grade 8 were requested to participate. Each school was requested to make available a copy of class lists. An appointment was scheduled with the school to deliver the consent forms for the children two weeks prior to the study and they were requested to return them within three days.

The study population consisted of 3764, children based on the numbers given by each school prior to data collection. Data were collected using the English version of ISAAC written and video questionnaires. Data on the latter, which were believed to be more specific for asthma, were not included in the analysis, as the questionnaire could not be completed in some schools, due to logistical problems such as unavailability of electricity, challenges of moving audio-visual equipment from class to class, or lack of a suitable venue where the children could watch the video.

### Health outcomes

In this study, we estimated the following health outcomes, on the basis of positive answers from the written questionnaire: wheeze ever [“Have you ever had wheezing or whistling in the chest at any time in the past?”], current wheeze [“Have you had wheezing or whistling in the chest in the past 12 months?”]. Current severe wheeze was defined as those who, according to the written questionnaire, responded positively to all four questions:

1) “How many attacks of wheeze have you had in the past 12 months?”. For this question, the children could select one of the following four options: a) none, b) 1–3 attacks, c) 4–12 attacks or d) more than 12 attacks in the past 12 months. Included in the definition of severe wheeze were those who had 4–12 attacks, or more than 12 attacks, in the past 12 months.

2) “In the past 12 months how often on average has your sleep been disturbed due to wheezing?”. For this question, the children could select one of the following three options: a) Never woken up with wheezing, b) one night per week, c) one or more nights per week. Included in the definition of severe wheeze were those who indicated one night per week or one or more nights per week.

3) “In the past 12 months, has wheezing ever been severe enough to limit your speech to only one or two words at a time between breaths?”. For this question, the option was yes or no.

4) “In the past 12 months, has your chest ever sounded wheezy during or after exercise?”. For this question, the option was yes or no.

### Air pollution sources

Air pollution sources included: ETS exposure at home in the past 30 days (yes/no), ETS exposure at school in the past 30 days (yes/no), tobacco smoking by participant (yes/no), mother/father smoking tobacco (yes/no), any other person smoking tobacco at home other than parents (yes/no). The children were asked to select the most frequently used source of energy at home, therefore had to select only one type of energy source: for cooking (electricity/gas/paraffin/wood/coal) and for heating (electricity/gas/paraffin/wood/coal). Other air pollution sources were: transportation mode to school (walking, informal taxi/bus, car, combination car/taxi or train), and frequency of trucks passing near residences (never/seldom/frequently through the day/almost all day).

### Confounders

Potential confounding variables included the following: sex (male/female), type of residential area (township: Tembisa or suburb: Kempton Park), period lived in the residential area (<6 months/6 to 12 months/1 to 2 years/≥3 years), type of house (brick/mud/corrugated iron/combination), availability of running water at home (yes/no), vigorous physical activity (never/occasionally/1-2 times per week/≥3 times per week), pets (cat and/or dog) in and around the home (yes/no), hours watching television per day (<1 hr/1 hr but <3 hrs/3 hrs but <5 hrs/≥5 hrs), use of paracetamol in the past 12 months (never/once per year/once per month), mother’s education level (primary school/secondary/tertiary education), average travel time from home to hospital (15 minutes’ walk or 5 minute drive/1 hour walk or 15 minutes’ drive/≥an hour’s walk or ≥ 30 minute drive), regular dietary intake of 15 food items e.g. meat, pasta, rice (never or occasionally/once or twice per week/three or more times a week).

### Data management and statistical analysis

The data were entered into a database set up in EpiInfo V3.5.3. Stata Version 12 was applied to the data analysis. The prevalence of the health outcomes and proportion of air pollution and confounding variables, were calculated by dividing the number of participants who responded affirmatively to a particular question by the number of questionnaires completed. Observations marked as “do not know”, “not stated” or “other responses” were set as missing. This resulted in each question having a slightly different sample size.

Crude and adjusted odds ratios (OR) and 95% confidence intervals (CI) were calculated to estimate the likelihood of health outcomes given the presence of an air pollution source and confounding variables. Univariate and multiple logistic regression analysis (LRA) were applied. All missing values were automatically excluded from the LRA. Air pollution and confounding variables that had *p* values estimated as less than 0.02 in the univariate LRA, were included in the multiple LRA. Only *p* values less than 0.05 in the multiple LRA, were considered statistically significant. The data were further stratified according to area of residence, Tembisa (township) or Kempton Park (suburb) to determine if the observed associations for the overall study population applied to the two areas separately. Interaction between vigorous physical activity and outdoor air pollution (i.e. truck traffic) were investigated to determine if there was any effect modification on current wheeze. During vigorous exercise more pollutants tend to be deposited in the lungs, resulting in increased bronchial hypersensitivity.

### Ethical considerations

This study was approved by the Ethics and Research Committee of the Faculty of Health Sciences, University of Pretoria (S121\2011). The Gauteng Department of Education, Ekurhuleni North District, the school principals and governing bodies were approached and gave their consent and cooperation for the study. Parents of participants were sent a letter explaining the details and nature of the study and were given the option of withdrawing their child from the study at any time should they wish to do so. Data collectors and capturers were instructed to keep all information confidential.

## Results

Of the 3764 children, 3468 completed the modified ISAAC questionnaire at the schools (92% response rate). The study focused only on those children who were present at the time of fieldwork and so 296 learners did not participate. The teachers gave an assurance that most of the children were present. School attendance was high during the study, thus bias that may have been introduced by non-response, was assumed to be relatively low. Forty-four questionnaires were excluded during data capture, due to incomplete information. A total of 3424 questionnaires were finally included in the data analysis.

The frequencies and percentages for general characteristics and living conditions are summarised in Table 1. The prevalence of having had wheeze in the past, current wheeze and current severe wheeze during the past 12 months was 32%, 18% and 12%, respectively. Girls accounted for 52% of participants and the mean age was 13 years. The majority of the children lived in Tembisa (67%). More than three quarters had lived in the same area for longer than three years (76%). Just over half of the children were born in the area where they were currently living. Nearly one third of the children reported occasionally or never engaging in any vigorous physical activity (29%). The majority exercised once or twice per week (42%), while the remaining third engaged in vigorous physical activity three or more times a week.

**Table 1:** **Demographic characteristics, health outcomes and sources of air pollution exposure of the study participants ( *n = 3424 *)**

**Variable**	**Total**	**Percentage**
**Sex of child**		
Female	1790	52.3
Male	1634	47.7
Missing	-	-
**Wheeze ever**		
Yes	1081	31.6
No	2343	68.4
Missing	-	-
**Current wheeze**		
Yes	619	18.2
No	2789	81.4
Missing	16	0.47
**Current severe wheeze**		
Yes	417	12.1
No	3007	87.8
Missing	-	-
**Residential area**		
Kempton Park	1117	32.6
Tembisa	2301	67.2
Missing	6	0.2
**Period lived in the residential area**		
<6 months	253	7.4
6 - 12 months	216	6.3
1 - 2 years	346	10.1
≥3 years	2609	76.2
Missing	-	-
**Mode of transport to schools**		
Walk	1728	50.5
Informal taxi\bus	708	20.1
Car	683	20
Combination car and informal taxi	201	5.9
Train	100	2.9
Missing	4	0.1
**Residential cooking fuel type most frequently used**		
Electricity	2995	87.5
Gas	179	5.2
Paraffin	200	5.8
Open fires (wood, coal)	30	0.9
Missing	20	0.6
**Residential heating fuel type most frequently used**		
Electricity	2041	59.6
Gas	426	12.4
Paraffin	631	18.4
Open fires (wood, coal)	270	7.9
Missing	56	1.6
**Frequency of trucks passing near homes on weekdays**		
Never	563	16.4
Seldom	1033	30.2
Frequently through the day	580	16.9
Almost all day	1212	35.4
Missing	36	1.1
**ETS exposure at school in the past 30 days**		
No	1452	42.4
Yes	1177	34.4
Missing	795	23.2
**ETS exposure at residence in the past 30 days**		
No	1460	42.6
Yes	1452	42.4
Missing	512	15
**Active tobacco smoker in the past 12 months**		
Yes	100	2.9
No	3274	95.6
Missing	50	1.5

Just over half of the children walked to school (51%), while the rest used other modes of transport (cars, taxi, buses and train). A small percentage of children reported gas most frequently (5%) and paraffin most frequently used (5%) for cooking at home, while the majority most frequently used electricity (88%). Twelve percent most frequently used gas for heating, 18% most frequently used paraffin, 7% most frequently used open fires (wood and coal) the remaining 52% most frequently used electricity. Ten percent of the children had a mother or female guardian who was a smoker, 27% a father or male guardian who was a smoker, or lived with someone other than their parents, who was a smoker (44%). Forty two percent of children reported having been exposed to ETS at home and 34% at school.

Other variables not included in Table 1 were measured as potential confounders. The majority (86%) of the children lived in formal housing structures and fewer than 20% lived in houses without running water. More than a quarter of the pupils spent, on average, more than five hours per day watching television (36%). Half of the children reported that the average travel time from home to a hospital was more than an hour’s walk or 30 minutes’ drive. More than one third of the children reported taking paracetamol at least once per year, while 44% reported taking it at least once per month during the past year. A small percentage (8%) reported having a cat at home in the past year, while 14% had had a cat in the past. A third of the children had a dog at home during the past year, while 43% had had one sometime in the past.

Tables 2, 3 and 4 summarise the results of multiple LRA for the overall study population. ETS exposure at school increased the likelihood of wheeze ever (OR 1.22 95% CI: 1.03 - 1.45) and current wheeze (OR 1.33 95% CI: 1.08 - 1.64). Gas most frequently used for heating was associated with wheeze ever (OR 1.47 95% CI: 1.15 - 1.88). Trucks passing near homes almost the whole day during weekdays increased the likelihood of wheeze ever (OR 1.32 95% CI: 1.01 - 1.73), current wheeze (OR 1.61 95% CI: 1.15 - 2.24) and current severe wheeze (OR 2.22 95% CI: 1.28- 3.77).

**Table 2:** Prevalence of wheeze ever amongst the participants (Tembisa and Kempton Park combined) along with crude and adjusted odd ratios

**Variable**	**Total**^**a**^	**Prevalence (%)**	**Crude OR (95% CI)**^**b**^	***p***	**Adjusted OR (95% CI)**^**b,c**^	***p***
**Sex of child**						
Female	1790	35.1	1		1	
Male	1634	27.7	0.7 (0.61-0.81)	**0.000**	**0.69 (0.58-0.82)**	**0.000**
**Residential area**						
Kempton Park	1117	37.1	1		1	
Tembisa	2301	28.9	0.69 (0.59-0.80)	**0.000**	**0.62 (0.51-0.76)**	**0.000**
**Vigorous physical activity per week**						
Never or occasionally	984	23.3	1		1	
Once or twice per week	1417	35.9	1.84 (1.53-2.22)	**0.000**	**1.66 (1.34-2.07)**	**0.000**
Three or more times a week	983	33.8	1.68 (1.37-2.05)	**0.000**	**1.39 (1.10-1.77)**	**0.006**
**Residential heating fuel type most frequently used**						
Electricity	2041	30.0	1		1	
Gas	426	39.2	**1.50 (1.21-1.86)**	**0.000**	**1.47 (1.15-1.88)**	**0.002**
Paraffin	631	31.8	1.06 (0.88-1.29)	0.508	0.06 (0.83-1.37)	0.597
Open fires (wood, coal)	270	33.3	1.16 (0.89-1.52)	0.261	1.16 (0.83-1.62)	0.360
**Frequency of trucks passing near homes on weekdays**						
Never	563	28.2	1		1	
Seldom	1033	33.3	1.26 (1.01-1.58)	0.038	1.10 (0.84-1.44)	0.351
Frequently through the day	580	28.4	1.01 (0.78-1.30)	0.938	0.89 (0.65-1.23)	0.539
Almost all day	1212	33.5	**1.27 (1.02-1.59)**	**0.020**	**1.32 (1.01-1.73)**	**0.035**
**ETS exposure at school in the past 30 days**						
No	1452	29.0	1		1	
Yes	1177	34.5	**1.20 (0.08-1.50)**	**0.004**	**1.22 (1.03-1.45)**	**0.020**

^a^Totals for each risk factor are different due to difference in missing value.

^b^Values that are statistically significant at less than 0.02 for the crude OR and less than 0.05 for the adjusted OR are in bold font.

^c^Model adjusted for all the variables in this table.

**Table 3:** Prevalence of current wheeze amongst the participants (Tembisa and Kempton Park combined) along with crude and adjusted odd ratios

**Variable**	**Total**^**a**^	**Prevalence (%)**	**Crude OR (95% CI)**^**b**^	***p***	**Adjusted OR (95% CI)**^**b,c**^	***p***
**Sex of child**						
Female	1779	20.8	1		1	
Male	1629	15.3	**0.69(0.57-0.82)**	**0.000**	**0.65 (0.53-0.81)**	**0.002**
**Residential area**						
Kempton Park	1114	21.9	1		1	
Tembisa	2290	16.3	0.69 (0.58-8.83)	0.000	0.61 (0.49-0.77)	0.000
**Vigorous physical activity per week**						
Never	980	13.6	1		1	
Once or twice per week	1409	20.7	**1.66 (1.33-2.08)**	**0.000**	**1.59 (1.22-2.07)**	**0.002**
Three or more time a week	979	19.4	**1.53 (1.20-1.95)**	**0.001**	**1.32 (0.99-1.77)**	**0.210**
**Use of paracetamol**						
Never	716	13.0	1		1	
At least once a year	1104	15.5	1.22 (0.93-1.61)		0.91 (0.65-1.26)	0.586
At least once per month	1523	22.5	**1.93 (1.51-2.48)**	**0.000**	**1.55 (1.14-2.09)**	**0.004**
**Frequency of trucks passing near homes on weekdays**						
Never	560	14.5	1		1	
Seldom	1029	19.0	1.38 (1.04-1.83)	0.025	1.24 (0.89-1.74)	0.151
Frequently through the day	580	16.4	1.15 (0.83-1.59)	0.371	0.97 (0.65-1.44)	0.874
Almost the whole day	1203	20.3	**1.50 (1.14-1.97)**	**0.004**	**1.61 (1.15-2.24)**	**0.006**
**Having a cat in and around the house in the past 12 months**						
No	3129	17.9	1		1	
Yes	264	21.6	**1.55 (1.23-1.95)**	**0.000**	**1.49 (1.13-1.98)**	**0.005**
**ETS exposure at school in the past 30 days**						
No	1446	16.2	1		1	
Yes	1169	20.2	**1.3 (0.06-1.59)**	**0.009**	**1.33 (1.08-1.64)**	**0.020**

^a^Totals for each risk factor are different due to difference in missing values.

^b^Values that are statistically significant at less than 0.02 for the crude OR and less than 0.05 for the adjusted OR are in bold font.

^c^Model adjusted for all the variables in this table.

**Table 4:** Prevalence of current severe wheeze amongst the participants (Tembisa and Kempton Park combined) along with crude and adjusted odd ratios

**Variable**	**Total**^**a**^	**Prevalence (%)**	**Crude OR (95% CI)**^**b**^	***p***	**Adjusted OR (95% CI)**^**b,c**^	***p***
**Pasta consumption**						
Never or occasionally	136	72.1	1		1	
Once or twice per week	319	67.1	0.81 (0.52-1.26))	0.359	0.78 (0.49-1.24)	0.301
Three or more times per week	122	59.0	**0.55 (0.33-0.93)**	**0.023**	**0.54 (0.31-0.93)**	**0.027**
**Vigorous physical activity per week**						
Never	133	59.4	1		1	
Once or twice per week	292	68.5	1.48 (0.97-2.27)	0.068	1.46 (0.93-2.30)	0.099
Three or more time a week	190	72.6	**1.81 (1.13-2.90)**	**0.013**	**1.76 (1.09-2.84)**	**0.030**
**Frequency of trucks passing near homes on weekdays**						
Never	81	56.8	1		1	
Seldom	195	62.1	1.24 (0.73-2.10)	0.416	1.16 (0.68-1.98)	0.598
Frequently through the day	95	67.4	1.57 (0.84-2.90)	0.150	1.44 (0.77-2.69)	0.333
Almost the whole day	244	75.4	**2.33 (1.37-3.95)**	**0.002**	**2.22 (1.28-3.77)**	**0.006**
**Having a cat in and around the house in the past 12 months**						
No	499	65.4	1		1	
Yes	117	76.1	**1.68 (1.06-2.67)**	**0.024**	**1.68(1.03-2.73)**	**0.035**

^a^Totals for each risk factor are different due to difference in missing values.

^b^Values that are statistically significant at less than 0.02 for the crude OR and less than 0.05 for the adjusted OR are in bold font.

^c^Model adjusted for all the variables in this table.

Significant confounders were gender, type of residential area and engaging in vigorous physical activity. Boys were found to be less likely to have wheeze ever (OR 0.69 95% CI: 0.58 - 0.82) and current wheeze (OR 0.65 95% CI: 0.53 - 0.81). Living in Tembisa significantly decreased the likelihood of wheeze ever (OR 0.62 95% CI: 0.51 - 0.76) and current wheeze (OR 0.61 95% CI: 0.49 - 0.77). Vigorous physical activity “once or twice a week” and “three or more times a week” increased the likelihood of wheeze ever (OR 1.66 95% CI: 1.34 - 2.07) and (OR 1.39 95% CI: 1.10 - 1.77), and current wheeze (OR 1.59 95% CI: 1.22 - 2.07) and (OR 1.32 95% CI: 0.99 - 1.77) respectively. Physical activity “three or more times a week” increased the likelihood of current severe wheeze (OR 1.76 95% CI: 1.09 - 2.84). In a sensitivity analysis, we investigated interaction between vigorous physical activity and truck traffic on current wheeze. We did not observe any interaction.

When the data were stratified by location of schools in Tembisa and Kempton Park (summarised in Tables 5 and 6), ETS exposure at home was associated with current wheeze for children residing in Tembisa (OR 1.36 95% CI: 1.06 - 1.77). In Tembisa gas most frequently used for residential heating was associated with wheeze ever, (OR 1.68 95% CI: 1.23 - 2.28) and current wheeze (OR 1.61 95% CI: 1.08 - 2.39). Paraffin most frequently for residential heating was associated with current severe wheeze (OR 1.85 95% CI: 1.04 - 3.28). In Kempton Park, gas most frequently used for residential cooking was associated with wheeze ever (OR 1.65 95% CI: 1.04 - 2.61). Trucks passing near homes almost the whole day during weekdays increased the likelihood of current wheeze for those residing in Kempton Park (OR 2.13 95% CI: 1.24 - 3.65) and the likelihood of current severe wheeze for those in Tembisa (OR 3.34 95% CI: 1.70 - 6.55).

**Table 5:** Prevalence of wheeze ever, current wheeze and severe wheeze amongst the participants from Tembisa along with crude and adjusted odd ratios

	**Total**^**a**^	**Prevalence (%)**	**Crude OR (95% CI)**^**b**^	***p***	**Adjusted OR(95% CI)**^**b**^	***p***
***Wheeze ever***^**c**^						
**Vigorous physical activity per week**						
Never or occasionally	696	20.8	1		1	1
Once or twice per week	975	40.1	**1.84 (1.47-2.31)**	**0.000**	**1.82 (1.44-2.28)**	**0.000**
Three or more times a week	605	32.4	**1.82 (1.41-2.33)**	**0.000**	**1.75 (1.36-2.26)**	**0.000**
**Residential heating fuel type most frequently used**						
Electricity	1233	26.7	1		1	
Gas	214	39.2	**1.77 (1.31-2.40)**	**0.000**	**1.68 (1.23-2.28)**	**0.001**
Paraffin	607	30.6	1.21 (0.98-1.50)	0.075	1.16 (0.94-1.45)	0.159
Open fires (wood, coal)	207	20.0	1.06 (0.77-1.48)	0.688	1.01 (0.73-1.41)	0.912
***Current wheeze***^**d**^						
**Residential heating fuel type most frequently used**						
Electricity	1225	15.2	1		1	
Gas	212	22.2	**1.59 (1.11-2.27)**	**0.011**	**1.61 (1.08-2.39)**	**0.018**
Paraffin	606	17.5	1.18 (0.91-1.53)	0.205	0.81 (0.58-1.13)	0.222
Open fires, wood, coal	207	31.0	0.83 (0.54-1.29)	0.424	0.63 (0.38-1.05)	0.077
**ETS exposure at home in the past 30 days**						
No	914	14.1	1		1	
Yes	998	18.7	**1.40 (1.09-1.79)**	**0.007**	**1.36 (1.06-1.77)**	**0.017**
**Vigorous physical activity per week**						
Never or occasionally	692	11.6	1		1	
Once or twice per week	970	18.8	**1.76 (1.33-2,34)**	**0.000**	**1.81 (1.31-2.50)**	**0.000**
Three or more times a week	603	18.2	**1.70 (1.25-2.33)**	**0.001**	**1.61 (1.12-2.30)**	**0.009**
***Severe wheeze***^**e**^						
**Residential heating fuel type most frequently used**						
Electricity	186	66.1	1		1	
Gas	47	78.2	1.89 (0.88-4.05)	0.100	1.95 (0.89-4.27)	0.093
Paraffin	106	79.2	**1.95 (1.11-3.42)**	**0.019**	**1.85 (1.04-3.28)**	**0.034**
Open fires, wood, coal	27	77.7	1.79 (0.68-4.66)	0.232	1.76 (0.66-4.68)	0.257
**Frequency of trucks passing near homes on weekdays**						
Never	52	55.7			1	
Seldom	80	90.2	1.64 (0.84-3.59)	0.131	1.90 (0.90-4.09)	0.088
Frequently through the day	61	70.1	1.89 (0.87-4.11)	0.107	2.02 (0.91-4.47)	0.080
Almost all day	180	80.55	**3.28 (1.69-6.35)**	**0.000**	**3.34 (1.70-6.55)**	**0.000**

^a^Totals for each risk factor are different due to difference in missing values.

^b^Values that are statistically significant at less than 0.02 for the crude OR and less than 0.05 for the adjusted OR are in bold font.

^c^Model adjusted for: Vigorous physical activity and residential heating fuel.

^d^Model adjusted for: Vigorous physical activity, residential heating fuel and ETS exposure at home.

^e^Model adjusted for: Residential heating fuel and frequency of trucks passing near homes.

**Table 6:** Prevalence of wheeze ever and current wheeze amongst the participants from Kempton Park along with crude and adjusted odd ratios

	**Total**^**a**^	**Prevalence (%)**	**Crude OR (95% CI)**^**b**^	***p***	**AdjustedOR (95% CI)**^**b**^	***p***
***Wheeze ever***^**c**^						
**Residential cooking fuel type most frequently used**						
Electricity	1003	35.1	1		1	
Gas	92	48.9	**1.73 (1.12-2.65)**	**0.012**	**1.65 (1.04-2.61)**	**0.030**
Paraffin	8	50.0	1.80 (0.44-7.27)	0.404	1.59 (0.37-6.70)	0.526
Open fires, wood, coal	10	50.0	1.80 (0.52-6.29)	0.351	1.83 (0.47-7.07)	0.375
**Residential heating fuel type most frequently used**						
Electricity	803	35.1	1		1	
Gas	211	39.3	1.28 (0.87-1.63)	0.256	1.06 (0.76-1.48)	0.696
Paraffin	24	50.0	1.84 (0.81-4.16)	0.139	1.61 (0.69-3.73)	0.267
Open fires, wood, coal	63	50.8	**1.90 (1.13-3.19)**	**0.014**	**1.75 (1.02-3.09)**	**0.040**
**Vigorous physical activity per week**						
Never or occasionally	228	29.1	1			
Once or twice per week	437	43.2	**1.85 (1.34-2.54)**	**0.000**	**1.80 (1.30-2.49)**	**0.000**
Three or more times a week	377	36.0	1.37 (0.95-1.90)	0.061	1.30 (0.93-1.83)	0.119
***Current wheeze***^**d**^						
**Frequency of trucks passing near homes on weekdays**						
Never	186	15.6	1		1	
Seldom	526	26.8	1.49 (0.95-2.34)	0.077	1.46 (0.90-2.37)	0.123
Frequently through the day	164	20.7	1.41 (0.81-2.44)	0.213	1.34 (0.74-2.44)	0.322
Almost all day	226	28.3	**2.13 (1.30-3.49)**	**0.002**	**2.13 (1.24-3.65)**	**0.006**
**Vigorous physical activity per week**						
Never or occasionally	288	18.4	**1**			
Once or twice per week	435	20.0	**1.48 (1.03 - 2.14)**	**0.024**	1.35 (0.90-2.20)	0.145
Three or more times a week	376	21.2	1.19 (0.81 - 1.18)	0.360	1.04 (0.67-1.60)	0.856
**ETS exposure at school in the past 30 days**						
No	507	19.7	**1**			
Yes	445	24.4	**1.74 (1.123-2.69)**	**0.013**	1.34 (0.98-1.60)	0.066

^a^Totals for each risk factor are different due to difference in missing values.

^b^Values that are statistically significant at less than 0.02 for the crude OR and less than 0.05 for the adjusted OR are in bold font.

^c^Model adjusted for: Residential cooking/heating fuel types and vigorous physical activity.

^d^Model adjusted for: Frequency of trucks passing near homes, vigorous physical activity and exposure to tobacco smoke at school.

Transportation mode to school, active smoking by study participant, mother/father smoking, or any other person smoking at home, were not significantly associated with wheeze by the univariate LRA.

## Discussion

The study investigated the association between wheeze, and indoor and outdoor air pollution sources, in an urban industrialised area in South Africa. When Tembisa and Kempton Park were considered together, detrimental associations were observed between wheeze ever/current wheeze/severe wheeze in 13–14 year old children, and exposure to ETS at school, residential gas heating and truck traffic near homes.

A recent report from centres in different countries that participated in the ISAAC Phase III, reported a global average of 14% for current wheeze for 13–14 year old children [16]. Current wheeze ranged from 5% in Northern and Eastern Europe to 22% in Oceania. Thirty-five centres (15%) had a prevalence of current wheeze ≥20%, located mostly in English language countries and Latin America. Twenty two centres (9%) had a prevalence of <5%, mostly in the Indian subcontinent, Asia-Pacific and eastern Mediterranean [21]. The prevalence of current wheeze reported in this study, was similar to that reported in English language countries and Latin America. Although these countries may have a high prevalence of asthma, the disease appears to be less often recognised and more severe in Africa, the Indian subcontinent and the Eastern Mediterranean [21]. The prevalence of current wheeze and current severe wheeze for the Polokwane ISAAC study was 11% and 6%, respectively [16], while the prevalence of current wheeze was 20% in the Cape Town study [15].

Exposure to ETS at school was positively associated with wheeze ever and current wheeze for the overall study population. Health effects associated with exposure to ETS have long been established [22-26]. A more recent literature review by Burke et al. reported that exposure to passive smoking increases the incidence of wheeze and asthma in children and young people by at least 20% [27]. Current legislation and policies on tobacco smoking should be strengthened to reduce smoking in public places such as schools. In the stratified analysis, ETS exposure at home was significant for current wheeze in Tembisa. Significantly more ETS exposure at home was observed for Tembisa than Kempton Park (chi square test < 0.0001). It is plausible that exposure to tobacco smoke might be associated with lower socio-economic status (SES) [28-31]. Eight percent of the children in Tembisa lived in informal housing structures (corrugated iron) compared to 0.7% in Kempton Park (chi square test p <0.0001). However, residual confounding due to other unmeasured SES factors may still be present.

Gas most frequently used for heating (overall study population) was only associated with wheeze ever. However, when the data were stratified by Tembisa and Kempton Park, it was interesting to observe that for children residing in Tembisa, gas most frequently used for heating at home was associated wheeze ever and current wheeze, whilst for those in Kempton Park gas most frequently used for cooking was associated with wheeze ever. Although current wheeze and severe wheeze (more specific outcomes than wheeze ever) were less common in Tembisa than Kempton Park, when gas or paraffin were most frequently used for residential heating the likelihood of these health outcomes increased in Tembisa, but not in Kempton Park. A reason for this may due to the fact that gas and paraffin are significantly more frequently used for residential heating in Tembisa than Kempton Park (chi square test p < 0.0001). Also, significantly more polluting fuels that were frequently used for cooking occurred in Tembisa than in Kempton Park (chi square test p < 0.0001).

Even though there is an increase in the electrification of both rural and urban areas, many South African households still rely on alternative sources of energy such as biomass fuel, gas and paraffin. According to the 2011 South African Census report, 26% of 51 million people still relied on alternative energy for cooking and 41.2% for heating. Eight and a half percent of the South African population still relied on paraffin for cooking and the same percentage applied for heating. This was due to rising costs of electricity. Gas heaters have been identified as a common indoor source of nitrogen dioxide (NO_2_) and particulate matter [8]. Heating devices can be used for many hours per day reflecting higher exposure, which over time can have cumulative health effects [32]. A study conducted by Muller et al., to assess the health risks of paraffin usage in an informal settlement in Durban, South Africa, showed a significant health risk resulting from paraffin usage in homes [9]. Venn et.al also found an association between wheeze and the use of paraffin in the home, in a study conducted in Ethiopia [33]. Ruiz et al. investigated the impact of gas and paraffin space heaters on indoor air quality in Chile and found an impact only for paraffin heaters [34].

For the overall study population, trucks passing near homes almost the whole day during weekdays, were identified as another air pollution source that had a detrimental association with wheeze ever, and an even stronger link to current wheeze and severe wheeze. It is well established that living close to a road with heavy traffic is associated with wheeze and other respiratory symptoms [35-37]. Brunekreef et al. analysed data from 110 ISAAC centres and found a positive relationship between symptoms of current wheeze in 13 to 14 year olds and self-reported truck traffic near homes [38]. A similar OR was observed for this study. When the data were stratified by area of residence, trucks passing near homes increased the likelihood of current wheeze for those in Kempton Park. The association was much stronger when compared to the unstratified analysis. The association between truck traffic and current severe wheeze for Tembisa was stronger than that of the overall sample. It appears that traffic-related air pollution is an important risk factor for children in both Tembisa and Kempton Park.

Although the majority of studies have been conducted in developed countries, a few studies from Africa have also found an association between wheeze and frequency of trucks passing near homes [39,40]. With an increase in industrialisation and number of vehicles, developing countries are facing the same challenges as those in developed countries, i.e. experiencing high levels of pollution, especially in urban areas where the majority of people are concentrated. In recent years South Africa has been developing rapidly and the number of cars on South African roads has increased tremendously, the number of vehicles (licensed only) in Gauteng province alone, in February 2013, was over 4.2 million [41]. As the number of vehicles continues to increase annually, traffic levels will increase on South African roads, leading to increased levels of traffic related pollution.

In a sensitivity analysis, interaction (effect modification) between vigorous physical activity and truck traffic on current wheeze was investigated. We did not observe any effect modification. None of the other ISAAC studies investigated effect modification between these two factors.

Limitations should be taken into account in the interpretation of the results. The ISAAC methodology has many inherent limitations: Firstly, the study had a cross-sectional epidemiological design. Secondly, the results were based on self-reported answers from a questionnaire. Self-reported answers can introduce recall bias, which may lead to misclassification of disease and exposure status. Children who had recently experienced episodes of wheezing, might have exaggerated their exposures, which may have led to overestimation, while underestimation could have occurred for those who did not recall exactly when wheezing occurred. Thirdly, the study found an association between air pollution sources and wheeze ever. The question on wheeze ever, does not mention attacks of wheezing in order to identify children with persistent symptoms characterised as episodes of attacks [42]. Many studies focus on current wheeze. Responses to questions about self-reported wheezing during the previous 12 months were shown to have good specificity for both bronchial hypersensitivity and a diagnosis of asthma in both children and adults [43]. Fourth, the exclusion of the video questionnaire data is disappointing, because it is believed to be more specific for asthma.

Fifthly, no quantitative air pollution exposure assessment was conducted during the study; the data were not analysed for mixed fuel types for residential heating or cooking, frequency and duration of fuel use at home and the number of cigarettes smoked were not included. Sixth, the frequency of trucks passing near homes on weekdays, may have been misclassified, as on weekdays, children are at school. Lastly, although the study area was done in an air pollution priority area, on the basis of multiple sources of air pollution; only proximity to truck traffic was investigated as an ambient (outdoor source) exposure variable. We did not include any other questions e.g. on distance of industries from residential areas. More research should be conducted in the area to investigate other outdoor air pollution sources.

The strength of our study is mainly the use of a validated ISAAC questionnaire regarding symptoms of wheeze. The ISAAC core questions have been used extensively in international studies of childhood asthma. Furthermore the participation rate was very high, which eliminated the risk of selection bias. This is the first ISAAC study conducted in an urban industrialised area in South Africa and the first to report an association between wheeze and traffic-related pollution. The study will contribute to existing literature about the prevalence of asthma symptoms amongst South African children, particularly of the age group 13 to 14 years. The baseline data will serve as a benchmark for future epidemiological studies.

## Conclusion

It was concluded that wheeze in children was associated with ETS, types of fuel used for residential cooking or heating and the frequency with which trucks passed close to homes in Ekurhuleni. Tobacco use was more strongly associated with wheeze in children exposed to smoking at school, than at home. It is advised that smoking exposure at schools needs to be better controlled. There appeared to be a difference between air pollution levels and wheeze, in children living in urban areas of Tembisa, in contrast to Kempton Park, which may be associated with different socioeconomic levels. It was also interesting that trucks passing close to homes in residential areas investigated were associated with more severe symptoms. This needs further investigation.

## Abbreviations

CI: Confidence intervals; BMF: Biomass fuel; EMM: Ekurhuleni Metropolitan Municipality; ETS: Environmental tobacco smoke; OR: Odds ratio; ISAAC: International Study of Asthma and Allergies in Childhood; NO2: Nitrogen dioxide; LRA: Logistic regression analysis.

## Competing interest

The authors declare that they have no competing interests.

## Acknowledgements

The authors would like to thank all the children who completed the questionnaires, the parents, school principals and the Gauteng Department of Education for giving permission to conduct the study, the students who conducted the interviews, the data capturers and Cornelius Nuttey for his assistance during the data processing stages.

The authors would like to thank the University of Pretoria, Tshwane University of Technology, Medical Research Council and the National Research Foundation for funding the study for academic research purposes.

## Authors’ contributions

JS participated in the design of the study, acquisition of data, statistical analysis and interpretation of the results and draft of the manuscript. JW participated in the design of the study, statistical analysis, interpretation of results and critically revised the manuscript. KV participated in the design of the study, statistical analysis, interpretation of results and critically revised the manuscript. All authors have read and approved the final manuscript.
